# Is premeiotic genome elimination an exclusive mechanism for hemiclonal reproduction in hybrid males of the genus *Pelophylax*?

**DOI:** 10.1186/s12863-016-0408-z

**Published:** 2016-07-02

**Authors:** Marie Doležálková, Alexandr Sember, František Marec, Petr Ráb, Jörg Plötner, Lukáš Choleva

**Affiliations:** Laboratory of Fish Genetics, Department of Vertebrate Evolutionary Biology and Genetics, Institute of Animal Physiology and Genetics CAS v.v.i, Liběchov, 277 21 Czech Republic; Department of Zoology, Faculty of Science, Charles University in Prague, Praha 2, 128 43 Czech Republic; Department of Genetics and Microbiology, Faculty of Science, Charles University in Prague, Viničná 5, Prague 2, 128 44 Czech Republic; Laboratory of Molecular Cytogenetics, Institute of Entomology, Biology Centre CAS, České Budějovice, 370 05 Czech Republic; Museum für Naturkunde, Leibniz Institute for Evolution and Biodiversity Science, Invalidenstraße 43, Berlin, 10115 Germany; Department of Biology and Ecology, Faculty of Science, University of Ostrava, Chittussiho 10, Ostrava, 710 00 Czech Republic

**Keywords:** Hybridogenesis, Asexual propagation, Hemiclone, Meiotic cycle, Genomic *in situ* hybridization, *Rana esculenta*

## Abstract

**Background:**

The ability to eliminate a parental genome from a eukaryotic germ cell is a phenomenon observed mostly in hybrid organisms displaying an alternative propagation to sexual reproduction. For most taxa, the underlying cellular pathways and timing of the elimination process is only poorly understood. In the water frog hybrid *Pelophylax esculentus* (parental taxa are *P. ridibundus* and *P. lessonae*) the only described mechanism assumes that one parental genome is excluded from the germline during metamorphosis and prior to meiosis, while only second genome enters meiosis after endoreduplication. Our study of hybrids from a *P. ridibundus*—*P. esculentus*-male populations known for its production of more types of gametes shows that hybridogenetic mechanism of genome elimination is not uniform.

**Results:**

Using comparative genomic hybridization (CGH) on mitotic and meiotic cell stages, we identified at least two pathways of meiotic mechanisms. One type of *Pelophylax esculentus* males provides supporting evidence of a premeiotic elimination of one parental genome. In several other males we record the presence of both parental genomes in the late phases of meiotic prophase I (diplotene) and metaphase I.

**Conclusion:**

Some *P. esculentus* males have no genome elimination from the germ line prior to meiosis. Considering previous cytological and experimental evidence for a formation of both *ridibundus* and *lessonae* sperm within a single *P. esculentus* individual, we propose a hypothesis that genome elimination from the germline can either be postponed to the meiotic stages or absent altogether in these hybrids.

**Electronic supplementary material:**

The online version of this article (doi:10.1186/s12863-016-0408-z) contains supplementary material, which is available to authorized users.

## Background

Meiosis is a vital process in all sexual organisms, ensuring fertility and genome stability and encouraging genetic diversity [14, 22]. Sexual reproduction involves the recombination of parental genomes followed by the coordinated segregation of the recombined chromosomes into gametes [57]. Despite the conservative nature of meiotic machinery, a number of anticipated mechanisms, including hybridization, can disrupt the regular cycles and alter the normal course of meiosis [41]. In hybrid animals, these deviations have resulted in a loss of sexual reproduction accompanied by modifications in gametogenesis such as premeiotic endomitosis (duplication of chromosomes), and genome exclusion (the loss of one parental genome) (reviewed in [26, 43]).

Hybridogenesis is a mode of bisexual reproduction characterized by the exclusion of one complete parental genome from the germline, while the remaining genome is endoreduplicated and subsequently transferred clonally (referred to as a hemiclone; [39, 55]). Hybridogenetic animals usually mate with the sexual species that contribute the eliminated genome [6, 9, 39]. New hybrids are generated via true fertilization, however, the genome from the sexual mate is discarded again in the next round of gamete formation.

Hybridogenesis has been recorded in the diploid all-female fish of the genus *Poeciliopsis* [39, 40], and Cimino [7, 8] observed the exclusion of *P. lucida* chromosomes during the onset of meiosis, while in *P. monacha* the genome is transferred into a reconstituted nucleus by the unipolar spindle. Apart from these species, very little is known about the cytological processes in other hybridogenetic or hybridogenesis-related animals such as the *Squalius alburnoides* fish [1], the *Misgurnus anguillicaudatus* fish [27], the Asian loach fish of the genus *Cobitis* [23], the carp gudgeon *Hypseleotris* [38], *Ambystoma* salamanders, *Bufotes baturae* toads [44], and *Pelophylax esculentus* water frogs [10, 17, 49].

The European sexual species *Pelophylax lessonae* and *P. ridibundus* hybridize and produce the hybrid form *P. esculentus*, which maintains a permanent F1 (first filial) hybrid state from generation to generation. This hybrid is able to exclude one parental genome from its germline and to duplicate the remaining one. As a result, the hybrid produces unrecombined *ridibundus* or *lessonae* gametes and therefore continues with only one parental species, i.e. the species whose genome has been eliminated (e.g. [2, 18, 47]).

It is generally believed that the exclusion of a parental genome from *P. esculentus* germ cells takes place before the onset of meiotic prophase I, followed by the endoreduplication of the remaining *ridibundus* genome [10, 11, 48]. In females the majority of oogonia have already been transformed into oocytes with 13 diplotene bivalents, usually by the time *P. esculentus* have entered their first hibernation [48]. Similarly, the proliferating spermatozoa in the testes of adult *P. esculentus* contained a diploid set of only *ridibundus* chromosomes [20]. Hence, the process of genome elimination and reduplication seems to occur at an early stage of spermatogenesis [20]. Further evidence comes from Günther [17], who observed in *P. esculentus* males from Eastern Germany a large number of meiotic figures with irregularities such as aneuploidy, univalency and heterologous multivalency. He interpreted his results as evidence contradicting the occurrence of a single cytological mechanism of hybridogenesis. Detailed cytological studies of male meiosis have yet to be carried out.

*P. esculentus* typically forms two reproductive systems; one with *P. lessonae* and one with *P. ridibundus*. The latter mostly consists of *P. ridibundus* (females and males) and only diploid hybrid males [50, 51]. Such *P. ridibundus*—*P. esculentus*-male populations have been found in Central Europe, mostly along the Oder River (reviewed by [34]). Here, hybrid males inherit either the *lessonae* or the *ridibundus* genome, or produce a combination of both kinds of sperm [3, 19, 35, 51, 54].

In order to understand the cytogenetic basis of these inheritance patterns, we studied the mitotic and meiotic cell stages of hybrids of a *P. ridibundus*—*P. esculentus*-male population from the Upper Oder River. Using comparative genomic hybridization (CGH) we discovered that the elimination of one parental genome does not necessarily precede meiotic divisions. In fact, the opposite is often true, where maintaining both parental genomes later in meiotic phases is actually relatively common.

## Methods

### Animals

We examined 14 adult and 4 subadult male individuals of *P. esculentus* from three different *P. ridibundus*—*P. esculentus* male populations along the Upper Oder River (49.914498, 18.091502; 49.705486, 18.092624; 49.735014, 18.152479). For genomic probes, we used two adult *P. lessonae* males (50.043063, 13.441079; 49.761259, 18.597399) and two adult *P. ridibundus* males from surrounding localities (49.705293, 18.081609). Specimens were genotyped using three polymorphic allozyme loci: Aspartate aminotransferase (*Aat*; EC 2.6.1.1), *Glucose-6-phosphate isomerase* (*Gpi*; EC 5.3.1.9) and Lactate dehydrogenase (*Ldh-1*; EC 1.1.1.27) [50]. All experimental procedures were conducted with the approval, and under the supervision of the Ethical Committee of the Faculty of Science,

Charles University, Prague, according to the directives of the State Veterinary Administration of the Czech Republic, permit number 34711/2010-30 from the Ministry of Agriculture of the Czech Republic. Specimens were deposited in the frog collection of the Laboratory of Fish Genetics, IAPG CAS, Liběchov. Permissions 358/2011 required for the field work collection of the frogs were obtained from the Agency for Nature Conservation and Landscape Protection of the Czech Republic.

### Chromosome preparations

We employed two different protocols to obtain chromosome spreads from gonadal tissues. In the majority of adult and subadult individuals we adapted the protocol of Zaleśna et al. [56], originally designed for chromosome preparation from bone marrow. In juvenile specimens with small gonads we applied a spreading technique previously used for spiders [25] with slight modifications. Briefly: after the dissection of a juvenile specimen the gonads were removed and hypotonized in 0.075 M KCl for 8 min, followed by three rounds (15, 30, 60 min) of fixation in 3:1 methanol / acetic acid solution. The fixed gonadal tissue was then suspended in 60 % acetic acid and spread on a hot-plate (40 °C).

For conventional cytogenetic analysis, chromosomes were stained with 5 % Giemsa solution (pH 6.8) (Merck, Darmstadt, Germany). Selected slides were destained in methanol / acetic acid fixative, dehydrated in an ethanol series (70, 80, and 96 %, 3 min each) and stored in a freezer (-20 °C) for subsequent cytogenetic experiments.

### DNA extraction and probe preparation

Whole genomic DNAs (gDNAs) from *P. ridibundus* and *P. lessonae* were extracted from muscle tissue using the conventional phenol-chloroform-isoamylalcohol method [13]. Probes prepared from both parental species were differentially labelled either with biotin-16-dUTP (2’-Deoxyuridine, 5’-Triphosphate, Roche, Mannheim, Germany) or digoxigenin-11-dUTP (Roche) using Nick Translation Mix (Abbott Molecular, Illinois, USA or Roche Diagnostics, Mannheim, Germany). For each slide, 1 μg of *P. ridibundus* gDNA, 1 μg of *P. lessonae* gDNA and 50 μg of sonicated salmon sperm DNA (Sigma-Aldrich) were added and the resulting probe was precipitated in 96 % ethanol, washed in 70 % ethanol, air-dried and re-dissolved in 25 μl of hybridization buffer (50 % formamide, 10 % dextran sulphate, 2× SSC (Standard saline buffer), 0.04 M NaPO_4_ (Sodium Phosphate) buffer, 0.1 % SDS, Denhardt’s reagent, see [29]). In some experiments, the final probe also included 15–30 μg of unlabelled species-specific competitive DNA prepared from *P. esculentus* gDNA using a Illustra GenomiPhi V2 DNA Amplification Kit (GE Healthcare, Buckinghamshire, UK), followed by sonication of the amplified product (40 cycles, 10 pulses, 100 % power) to approximate fragment size of 100–200 bp using the ultrasonic homogenizer Sonopuls HD 2070 (Bandelin Electric, Berlin, Germany).

### Comparative genomic hybridization (CGH)

In order to identify the chromosome sets of particular parental species within a hybrid genome throughout the meiotic phases we performed the CGH method according to Bi and Bogart [4] with several modifications. After thermal aging (3–4 h at 37 °C and 1 h at 60 °C) the chromosomes were treated with RNase A (Sigma-Aldrich) (200 μg/ml in 2× SSC, 90 min, 37 °C) and then pepsin (50 μg/ml in 10 mM HCl, 3 min, 37 °C). The slides were denatured in 75 % formamide (pH 7.0) (Sigma-Aldrich) in 2× SSC at 74 °C for 3 min, and then immediately cooled and dehydrated in 70 % (cold), 80 % and 96 % (RT) ethanol. The hybridization mixture was denatured at 86 °C for 6 min. Hybridization was performed at 37 °C for 48–72 h. Post-hybridization washes were applied twice in 50 % formamide in 2× SSC (pH 7.0) at 42 °C for 5 min and three times in 1× SSC at 42 °C (7 min each). In order to block non-specific binding sites for streptavidin and anti-digoxigenin, the slides were incubated with 500 μl of 3 % BSA (Vector Labs, Burlington, Canada) in 4× SSC in 0.01 % Tween 20 at 37 °C for 20 min. The hybridization signal was detected using Anti-Digoxigenin-Rhodamine (Roche) and Streptavidin-FITC (fluorescein isothiocyanate; Invitrogen Life Technologies, San Diego, CA, USA) or alternatively with Anti-Digoxigenin-Fluorescein (Roche) and Streptavidin-Cy3 (Invitrogen Life Technologies), to exclude any influence of antibodies and/or fluorochromes. The slides were incubated with antibodies at 37 °C for 60 min in a dark humid chamber. Finally, the slides were washed four times (7 min each) in 4× SSC in 0.01 % Tween (pH 7.0) at 42 °C and mounted in antifade containing 1.5 μg/ml DAPI (4’, 6-diamidino-2-phenylindole; Cambio, Cambridge, United Kingdom).

### Image processing

Chromosomal preparations were inspected using a Provis AX70 (Olympus) fluorescence microscope equipped with standard fluorescence filter sets. Selected images for each fluorescent dye were captured separately with a black and white CCD camera (DP30BW Olympus) using Olympus Acquisition Software. The digital images were then pseudocoloured (blue for DAPI, red for Rhodamine or Cy3, green for FITC) and superimposed using MicroImage software (Olympus, version 4.0). The images were optimized for brightness and contrast using Adobe Photoshop, version CS5.

## Results

**Fig. 1:**
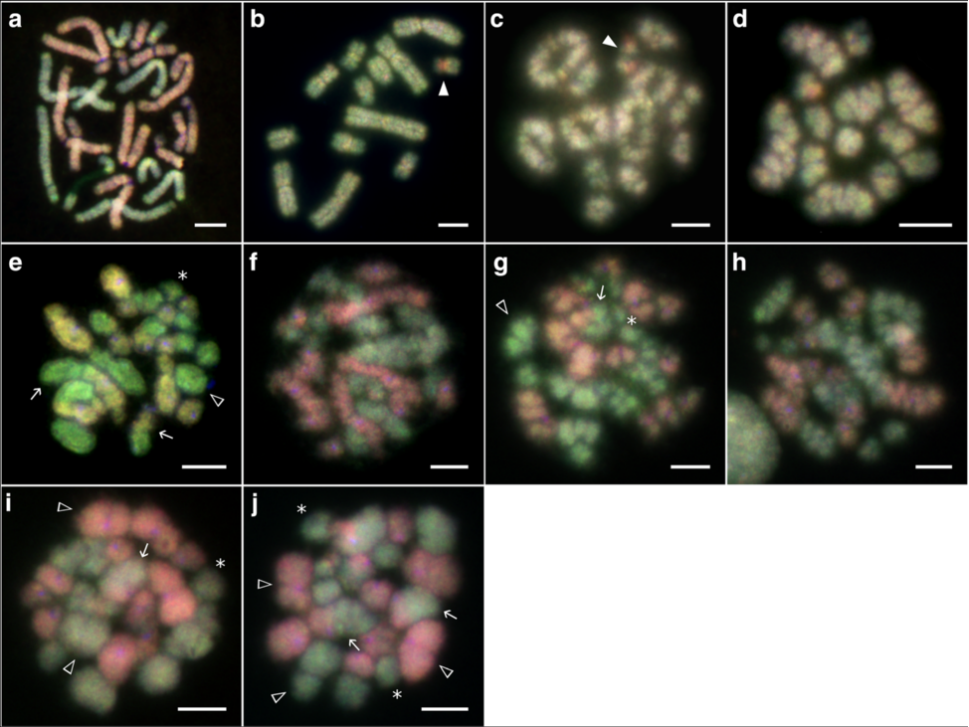
Comparative genomic hybridization (CGH) in mitotic and meiotic chromosomes of four water frog *Pelophylax esculentus* males. M1 (**a**), M2 (**b-d**), M3 (**e-g, j**) and M4 (**h, i**). CGH clearly distinguished chromosomes of the parental species, *P. ridibundus* (*red*) and *P. lessonae* (*green*). **a** Mitotic prometaphase. **b** Haploid mitotic metaphase after elimination of the *ridibundus* genome. **c** Diplotene. **d** Meiotic metaphase I. **e, f, g, h** Late meiotic prophase I. **i, j** Meiotic metaphase I showing bivalent-like configurations and univalents. *Solid arrowheads* indicate the smallest submetacentric chromosome pair with marked *ridibundus*-specific repetitive DNA in the *lessonae*-derived chromosome set, *arrows* indicate bivalent-like configurations between two different parental genomes, *open arrowheads* indicate bivalent-like configurations within one parental genome, asterisks indicate univalents. Scale bars equal 10 μm

We obtained chromosomal preparations from the gonads of 18 male individuals. The preparations contained different phases of meiotic division as well as spermatogonial mitotic metaphases. Giemsa-stained karyotypes (not shown) confirmed the previous description of Zaleśna et al. [56], with all species of the *Pelophylax* hybridogenetic complex having 26 metacentric and submetacentric chromosomes. Moreover, in line with the findings from the mentioned study, the homologous chromosomes in *P. esculentus* differed slightly in size. Along with spermatogonial metaphases, we also observed stages with haploid or diploid chromosome numbers corresponding to particular meiotic and/or pre-meiotic phases (Fig. 1a-e). Haploid chromosome complements appeared to correspond to either a premeiotic stage after the elimination of one parental genome (Fig. 1b) or to chromosomes in the first meiotic division (Fig. 1d). Diploid chromosome complements represented either mitotic metaphases (Fig. 1a) or stages of the first meiotic division with bivalents (Fig. 1c).

**Fig. 2:**
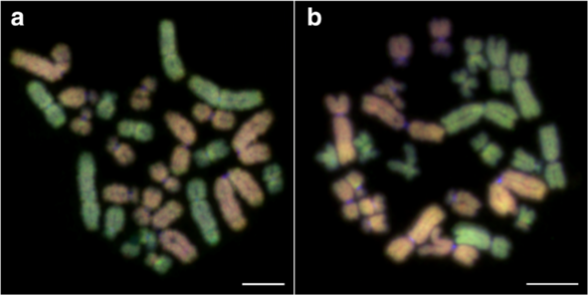
Mitotic metaphases of a *Pelophylax esculentus* male after comparative genomic hybridization (CGH). **a** CGH with specific competitive DNA prepared from *P. esculentus*. **b** CGH without specific competitive DNA. *P. ridibundus* chromosomes are visible as *red* signals, *P. lessonae* chromosomes as *green* signals. Scale bars equal 10 μm

We examined the mitotic and meiotic spreads further by means of CGH in four hybrid males (M1-M4). Although chromosome spreads were successfully obtained from all individuals, the hybridization procedure was only successful in four of them. Some examples of unsuccessful hybridization patterns are shown in Additional file 1: Figure S1-S3. A possible explanation for the general failure of CGH could be its high sensitivity in respect to experimental conditions [45, 46]. Multiple successful repetitions of the CGH experiments did however confirm that the chromosomal patterns observed in germinal cells of four *esculentus* males (M1-M4) were not artefacts. CGH provided a clear discrimination between the chromosomes of *P. lessonae* and *P. ridibundus* (Fig. 1a). The observed differential hybridization pattern of chromosome complements containing both parental genomes most probably resulted from the presence of species-specific repetitive sequences [24], very likely including some sort of transponable elements (TEs) and microsatellites [33]. Both experimental approaches (either with- or without the specific competitive DNA prepared from *P. esculentus*) yielded the same resulting hybridization pattern (Fig. 2a, b).

Two groups of males were distinguishable by their differences in hybridization patterns. In the first group (male M2), nearly all chromosomes, with the exception of the smallest submetacentrics, were predominately highlighted with the *lessonae*-derived probe (Fig. 1b-d). The smallest submetacentric chromosome pair displayed a marked *ridibundus*-specific repetitive DNA region, even in the homologous *lessonae*-specific chromosomes (Fig. 1b, c, *solid arrowheads*). The number and morphology of the chromosomes indicated the presence of both mitotic (Fig. 1b) and meiotic stages (Fig. 1c, d). In the second group, 89 out of 122 chromosome complements (49 out of 55 in male M1, 18/22 in male M3, and 22/45 in male M4) showed a mixture of chromosomes with two different hybridization patterns, i.e. with strong hybridization signals of the *lessonae*-derived probe and the *ridibundus*-derived probe (Fig. 1a, e-j). All chromosomal complements showing both parental genomes were classified as diploid sets, either composed of mitotic chromosomes (Fig. 1a) or meiotic chromosomes in a late meiotic prophase I (Fig. 1e, f, g, h) or in a metaphase I (Fig. 1i, j).

**Fig. 3:**
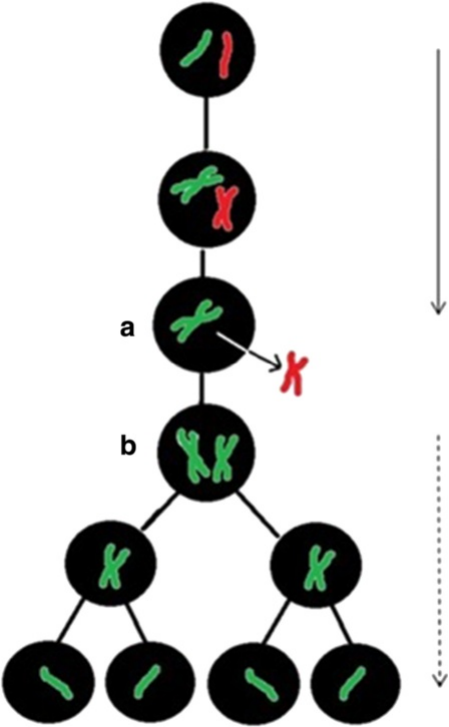
Schema of hybridogenesis assumed for maintenance of diploid hybrid male M2 (this study) in mixed populations with *P. ridibundus*. **a** elimination of the *P. ridibundus* genome (*red*); **b** reduplication of the *P. lessonae* genome (*green*). As a result haploid *P. lessonae* gametes are produced. The vertical solid arrow shows spermatogonia, the dashed arrow spermatocytes. Meiotic cycle starts after **b**

Based on the accurate identification of meiotic stages and on the scheme of hybridogenesis (Fig. 3) we tried to provisionally reconstruct the process of hybrid spermatogenesis. From 170 observed figures we identified five different mitotic or meiotic stages i.e. (i) mitotic metaphase with either diploid (Fig. 1a) or haploid (Fig. 1b) chromosome numbers, (ii) meiotic diplotene with regular bivalents (Fig. 1c) and (iii) meiotic metaphase MI (Fig. 1d) where 1c and 1d are composed of only one parental genome, (iv) late meiotic prophase I (Fig. 1e, f, g, h) and (v) meiotic metaphase MI (Fig. 1i, j) where chromosomes of both parental species formed bivalent-like configurations. More specifically, while male M2 exhibited only the *lessonae*-derived chromosomes in meiotic prophase I and metaphase I with 13 bivalents (each of them presumably composed of a pair of endoreduplicated identical chromosomes), the males M3 and M4 displayed chromosomes apparently derived from both parental genomes in their meiotic prophase I. These males formed bivalent-like configurations from non-homologous chromosomes that paired randomly either within (Fig. 1e, g, i, j, *open arrowheads*) or between parental genomes (Fig. 1e, g, i, j, *arrows*). Moreover, some chromosomes did not form a bivalent-like configuration, but instead remained unpaired as univalents (Fig. 1e, g, i, j, asterisks).

## Discussion

Our analysis of the meiotic mechanism of *Pelophylax esculentus* males provides supporting evidence of premeiotic genome elimination. In addition to this observation, we record the presence of both parental genomes in the late phases of meiotic prophase I (diplotene) and metaphase I in several other males. Our results suggest that some males have no genome elimination from the germ line prior to meiosis.

The formation of clonal gametes during hybridogenetic spermatogenesis depends on a range of coordinated molecular and cytogenetic processes that are not yet fully understood. It is generally believed that in the germ cells of diploid hybrids one parental chromosome set is eliminated before entering the meiotic cycle, while the remaining set is endoreduplicated (e.g., [20]). This pattern was observed in at least one hybrid male (M2; Fig. 1b-d). The meiotic divisions obtained from this male contained only green coloured *lessonae* chromosomes either in a haploid set, after the elimination of the red coloured *ridibundus* chromosomes, Fig. 1b), or in a diploid number, after genome duplication (Fig. 1c-d). Such an inheritance mode would lead to sperm with a *lessonae* genome, which would mean that after fertilization of the *P. ridibundus* egg the F1 hybrid state would be restored. As the meiotic chromosomes treated with comparative genomic hybridization (CGH) did not display any recombination between the *lessonae* and *ridibundus* chromosomes such as crossing-over or other types of recombination, this male must have transferred its *lessonae* genome clonally into its sperm as assumed for hybrid males from *P. ridibundus*—*P. esculentus*-male populations [19, 51].

A completely different pattern of spermatogenesis was found in males M3 and M4 where the majority of nuclei in the first meiotic division contained both *ridibundus* and *lessonae* chromosome sets. Most of the nuclei were in the late meiotic prophase I, probably corresponding to diplotene (Fig. 1e, f, g, h) with some of them even reaching metaphase I (Fig. 1i, j). This finding clearly suggests that the majority of spermatocytes did not carry out genome elimination prior to meiosis. Previous studies based on protein electrophoresis have indicated that in the germ line of *P. esculentus* genome elimination takes place before meiosis [12, 20, 52], likely during the last mitotic division [48] in the so called “E” (Elimination) phase [53]. There are two principle hypotheses concerning genome exclusion: 1) an exclusion takes place during the mitotic phase whereby the excluded genome is enzymatically degraded [31, 54], or 2) the elimination of whole chromosomes, or at least parts of them, takes place during mitosis of the gametogonia [31]. The latter hypothesis seems less likely as no irregularities in the spindle apparatus or in the heterochromatization have been observed (see pp. 91–92 of [34]). It is not yet clear whether genome elimination is a one-step or a gradual process during mitotic division [31]. Within vertebrates, only the all-female fish of the genus *Poeciliopsis* eliminate one chromosome set as late as in meiosis but even in this fish it occurs during prophase I [7, 8].

The occurrence of both parental genomes in the proliferating spermatozoa of *P. esculentus* investigated in this study conflicts with our expectation of observing only one parental genome in the meiotic cells of adult males [20]. It further suggests that the elimination phase (if present) is not restricted to the period around metamorphosis.

Using conventional cytogenetic techniques, the absence of genome exclusion has been assumed in some hybrids from *P. ridibundus*—*P. esculentus*-male populations [17, 21] and in just a single laboratory-synthesized *P. esculentus* male [36]. The related observations of numerous aberrations during meiosis in *P. esculentus* males such as aneuploidy, degenerated chromosomes and heterologous multivalents [17, 32] and of fertility disorders in many *P. esculentus* males (e.g. [15, 16, 30]) can be considered as evidence for selection processes acting during pregametic and/or gametic stages [19]. As well as cell lineages in which one parental genome is excluded premeiotically, lineages (spermatogonia, spermatocytes) with both parental genomes may undergo cellular selection during meiosis. As a result, lineages with balanced genomes (probably with the chromosomes of only one parental species) may yield fertile sperm while those with unbalanced haploid genomes (a mixture of *lessonae* and *ridibundus* chromosomes) would result in infertile sperm [19].

Indeed, irregular diplotene stages (Fig. 1e, g, i, j) with bivalent-like configurations and univalents, and the fact that most *ridibundus* chromosomes paired with non-homologous *ridibundus* chromosomes rather than with homologous *lessonae* chromosomes and vice-versa, may indicate malfunctions in the process of genome haploidization and meiosis in general. But in terms of the number of chromosomes, meiotic prophase I with 13 *ridibundus* and 13 *lessonae* chromosomes (Fig. 1i, j) did not differ from regular meiotic phases with 13 bivalents. More thorough analyses are necessary to understand whether such cells may or not produce functional sperm. Currently, two alternative hypotheses remain open. First, such cells may still result in dysfunctional sperms [19]. It was already observed that many *P. esculentus* males exhibit degenerated testes, low numbers of sperm, high numbers of immobilized and/or inhibited sperm [19, 30, 37]. Second, the cells may yield both unrecombined *lessonae* and *ridibundus* sperm [19, 51, 54]. Vinogradov et al. [54] recorded “so-called hybrid amphispermy” in 14–17 % of *P. esculentus* males. Although the underlying cytogenetic mechanisms were not identified, in principle, two mechanisms are conceivable: 1) genome exclusion is unspecific and takes place during meiosis leading to clonal cell lineages with only *lessonae* or *ridibundus* chromosomes, or 2) the chromosomes are segregated non-randomly during meiosis, probably in anaphase I, i.e. without interchromosomal recombination, resulting in both *lessonae* and *ridibundus* spermatids and sperms.

Chromosomal studies of deviations from canonical gametogenesis in *P. esculentus* females have shown observations of very rare oocytes in which elimination has not occurred [5, 10] resembling the mechanism of premeiotic endoreplication in automictic parthenogenesis [28, 42]. Dedukh et al. [10] also observed aneuploid oocytes suggesting a partial loss of chromosomes during gametogenesis. Together with our observations that some diploid *P. esculentus* males have no genome elimination from the germ line prior to meiosis, the phenomenon of no chromosome elimination may be more common than previously thought.

## Conclusions

The central finding of this study is that genome elimination in *P. esculentus* males is not always restricted to larval or juvenile stages, as both parental genomes were discovered to still be present in the germline of the adult specimens. We propose the following three hypotheses about the fate of homologous and non-homologous bivalent-like configurations of *lessonae* and *ridibundus* chromosomes observed in the first meiotic division: 1) such bivalents represent a process leading to unviable gametes; 2) the elimination phase is postponed to later stages of the meiotic cell cycle; 3) there is no genome elimination, homologous *lessonae* and *ridibundus* chromosomes segregate in anaphase I resulting in both haploid *lessonae* and *ridibundus* sperm.

Overall, our data provide new information about the behavior of two species-specific genomes in the meiotic cycle which will help us understand the underlying cytogenetic mechanisms regulating the formation of clonal gametes. As the molecular mechanisms leading to genome exclusion and subsequent gamete formation are still unclear, not only in water frogs but also in other asexuals, further research should focus on the mechanisms of homologous chromosome pairing and segregation in later meiotic phases.

## Abbreviations

*Aat*, aspartate aminotransferase; Cy3, cyanine dye; CGH, comparative genomic hybridization; DAPI, 4’, 6-diamidino-2-phenylindole; dUTP, 2’-Deoxyuridine, 5’-Triphosphate; E, elimination; F1, first filial generation; FITC, fluorescein isothiocyanate; gDNA, whole genomic DNA; *Gpi*, Glucose-6-phosphate isomerase; HCl, hydrogen chloride; IAPG CAS, v.v.i., Institute of Animal Physiology and Genetics of the Czech Academy of Sciences, v.v.i.; KCl, kalcium chloride; *Ldh-1*, lactate dehydrogenase; NaPO_4_, sodium phosphate; SDS, sodium dodecyl sulfate used as Denhardt’s reagent; SSC, Standard saline buffer; TEs, transposable elements 

## Additional file

Additional file 1: Figure S1-S3. Comparative genomic hybridization (CGH) on mitotic (1) and meiotic (2, 3) chromosomes of *Pelophylax esculentus* males showing several types of experimental artefacts and failures. 1) Unsuccessful differentiation of parental chromosomes: note the apparent accumulation of probes on the edges/surface of chromosomes, possibly due to over fixed gonadal tissues used for chromosome spreads. 2) Inconclusive hybridization pattern: note equal hybridization intensity of both genome-derived probes. 3) Week hybridization pattern, insufficient for differentiation of parental chromosomes. *Lessonae*-derived genomic probes were labelled with biotin-16-dUTP and hybridization signals detected with Streptavidin-FITC (*green*) (**1a**, **2a**, **3a**), *ridibundus*-derived genomic probes (**b**) with digoxigenin-11-dUTP and Anti-Digoxigenin-Rhodamine (*red*) (**1b**, **2b**, **3b**). Figures **1c**, **2c**, **3c** show merged images of both genomic probes, figures 1d, 2d, 3d merged images of both probes and DAPI staining of chromosomes (*blue*). Scale bar = 10 μm. (TIF 2427 kb)
