# Supplementary figures and images for: Is premeiotic genome elimination an exclusive mechanism for hemiclonal reproduction in hybrid males of the genus Pelophylax?
Source: BMC Genet. 2016 Jul 2;17:100. doi: 10.1186/s12863-016-0408-z (PMC4930623; doi:10.1186/s12863-016-0408-z)

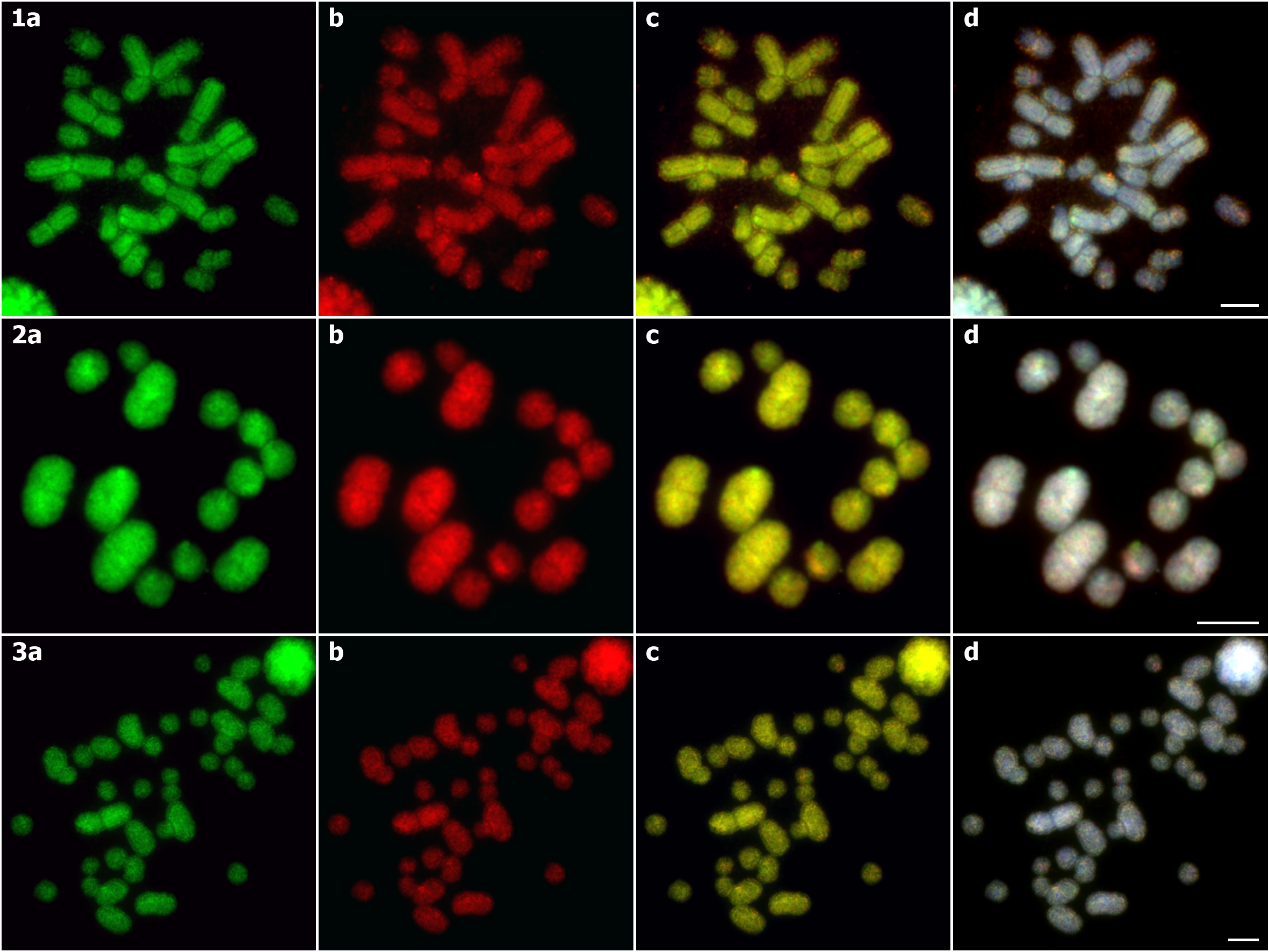

Supplement: Additional file 1: Figure S1-S3. — Comparative genomic hybridization (CGH) on mitotic (1) and meiotic (2, 3) chromosomes of Pelophylax esculentus males showing several types of experimental artefacts and failures. 1) Unsuccessful differentiation of parental chromosomes: note the apparent accumulation of probes on the edges/surface of chromosomes, possibly due to over fixed gonadal tissues used for chromosome spreads. 2) Inconclusive hybridization pattern: note equal hybridization intensity of both genome-derived probes. 3) Week hybridization pattern, insufficient for differentiation of parental chromosomes. Lessonae-derived genomic probes were labelled with biotin-16-dUTP and hybridization signals detected with Streptavidin-FITC (green) (1a, 2a, 3a), ridibundus-derived genomic probes (b) with digoxigenin-11-dUTP and Anti-Digoxigenin-Rhodamine (red) (1b, 2b, 3b). Figures 1c, 2c, 3c show merged images of both genomic probes, figures 1d, 2d, 3d merged images of both probes and DAPI staining of chromosomes (blue). Scale bar = 10 μm. (TIF 2427 kb) [file 12863_2016_408_MOESM1_ESM.tif]
